# 两个遗传性纤维蛋白原缺陷症家系表型与基因突变分析

**DOI:** 10.3760/cma.j.issn.0253-2727.2023.11.008

**Published:** 2023-11

**Authors:** 恺琦 贾, 正仙 苏, 荟琳 陈, 晓勇 郑, 蔓霖 曾, 柯 张, 龙颖 叶, 丽红 杨, 艳慧 金, 明山 王

**Affiliations:** 温州医科大学附属第一医院医学检验中心，浙江省检验诊断及转化研究重点实验室，温州 325015 Department of Clinical Laboratory, Key Laboratory of Clinical Laboratory Diagnosis and Translational Research of Zhejiang Province, The First Affiliated Hospital of Wenzhou Medical University, Wenzhou 325015, China

**Keywords:** 纤维蛋白原缺陷症, 基因突变, 纤维蛋白原, 蛋白模型, Fibrinogen deficiency, Gene mutation, Fibrinogen, Protein model

## Abstract

**目的:**

对两个杂合突变导致遗传性纤维蛋白原缺陷症家系进行表型和基因突变分析，并初步探讨其分子致病机制。

**方法:**

采用凝固法检测两个先证者及其各自家系成员（3代9人和2代3人）凝血酶原时间（PT）、活化部分凝血活酶时间（APTT）、凝血酶时间（TT）和纤维蛋白原活性（Fg∶C），免疫比浊法检测纤维蛋白原抗原（Fg∶Ag）。DNA直接测序法分析先证者FGA、FGB和FGG基因所有外显子和侧翼序列及家系成员相应的突变位点区域。通过凝血酶诱导进行纤维蛋白原聚集试验；用ClustalX-2、1-win软件分析突变位点的保守性；用Mutation Taster、PolyPhen-2、PROVEAN、SIFT和LRT在线生物信息学软件预测突变位点的致病性；用Swiss-pdb Viewer4.0.1分析突变前后蛋白质空间结构及分子作用力的变化。

**结果:**

家系1先证者和家系2先证者Fg∶C明显下降（分别为1.28 g/L、0.98 g/L）；家系1先证者Fg∶Ag正常（2.20 g/L），家系2先证者Fg∶Ag降低（1.01 g/L）。基因分析发现，家系1先证者的FGB基因第2号外显子存在c.293C>A（p.BβAla98Asp）杂合错义突变；家系2先证者的FGB基因第8号外显子存在c.1418C>G（p.BβSer473*）杂合无义突变。同源性分析表明Ala98和Ser473残基在同源物种间呈不同保守状态；在线生物信息学软件预测显示p.BβAla98Asp和p.BβSer473*突变为致病突变；蛋白模型分析显示，p.BβAla98Asp突变使氨基酸之间的氢键发生改变，p.BβSer473*突变产生了截短蛋白。

**结论:**

家系1先证者的异常纤维蛋白原血症和家系2先证者的低纤维蛋白原血症可能分别与p.BβAla98Asp杂合错义突变及p.BβSer473*杂合无义突变有关。

## Abstract (en)

**Objective:**

To analyze the phenotype and genotype of two pedigrees with inherited fibrinogen（Fg）deficiency caused by two heterozygous mutations. We also preliminarily probed the molecular pathogenesis.

**Methods:**

The prothrombin time (PT), activated partial thromboplastin time (APTT), thrombin time (TT) and plasma fibrinogen activity (Fg∶C) of all family members (nine people across three generations and three people across two generations) were measured by the clotting method. Fibrinogen antigen (Fg:Ag) was measured by immunoturbidimetry. Direct DNA sequencing was performed to analyze all exons, flanking sequences, and mutated sites of FGA, FGB, and FGG for all members. Thrombin-catalyzed fibrinogen polymerization was performed. ClustalX 2.1 software was used to analyze the conservatism of the mutated sites. MutationTaster, PolyPhen-2, PROVEAN, SIFT, and LRT online bioinformatics software were applied to predict pathogenicity. Swiss PDB Viewer 4.0.1 was used to analyze the changes in protein spatial structure and molecular forces before and after mutation.

**Results:**

The Fg∶C of two probands decreased (1.28 g/L and 0.98 g/L, respectively). The Fg∶Ag of proband 1 was in the normal range of 2.20 g/L, while it was decreased to 1.01 g/L in proband 2. Through genetic analysis, we identified a heterozygous missense mutation (c.293C>A; p.BβAla98Asp) in exon 2 of proband 1 and a heterozygous nonsense mutation (c.1418C>G; p.BβSer473*) in exon 8 of proband 2. The conservatism analysis revealed that Ala98 and Ser473 presented different conservative states among homologous species. Online bioinformatics software predicted that p.BβAla98Asp and p.BβSer473* were pathogenic. Protein models demonstrated that the p.BβAla98Asp mutation influenced hydrogen bonds between amino acids, and the p.BβSer473* mutation resulted in protein truncation.

**Conclusion:**

The dysfibrinogenemia of proband 1 and the hypofibrinogenemia of proband 2 appeared to be related to the p.BβAla98Asp heterozygous missense mutation and the p.BβSer473* heterozygous nonsense mutation, respectively. This is the first ever report of these mutations.

遗传性纤维蛋白原缺陷症（CFD）是指由于纤维蛋白原基因FGA、FGB、FGG缺陷导致纤维蛋白原含量/结构异常的一种遗传性疾病，呈常染色体隐性、显性或共显性遗传[1]。CFD可分为两型：①Ⅰ型缺陷主要是量的异常，表现为纤维蛋白原含量降低，包括无纤维蛋白原血症及低纤维蛋白原血症；②Ⅱ型缺陷主要是质的异常，表现为纤维蛋白原功能异常，包括异常纤维蛋白原血症及低且异常纤维蛋白原血症[2]。低纤维蛋白原血症患者多无临床表现；异常纤维蛋白原血症的临床表现呈现明显的异质性，55％无临床表现，25％表现为出血，20％表现为血栓形成倾向（多为静脉血栓）[3]。本研究对两个CFD家系进行表型及基因型分析，初步探讨其致病机制。

## 对象与方法

1. 家系资料：家系1先证者，女，48岁，浙江温州人，因“腹部不适数月”至我院就诊，消化内镜检查前凝血功能筛查示凝血酶时间（TT）为22.5 s（参考值14.0～20.0 s）、纤维蛋白原活性（Fg∶C）1.28 g/L（参考值2.00～4.00 g/L）、纤维蛋白原抗原（Fg∶Ag）2.20 g/L（参考值2.00～4.00 g/L），其他凝血指标未见异常。患者平素体健，无自发性出血及血栓病史。其他家系成员（共3代9人）均无自发性出血及血栓病史。家系图见图1。

**图1:**
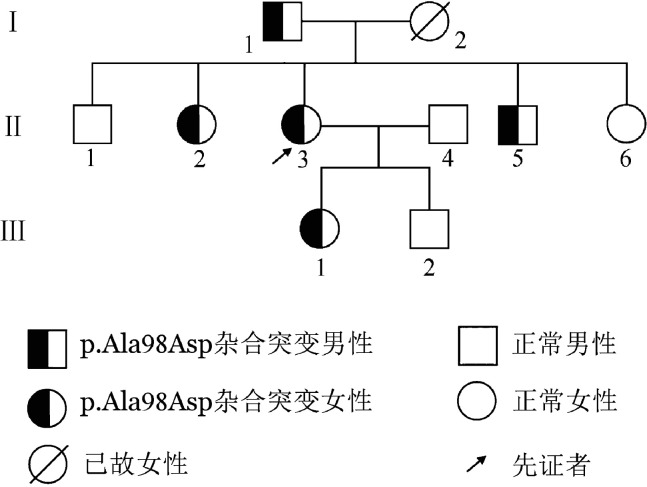
遗传性纤维蛋白原缺陷症（家系1）家系图

家系2先证者，男，29岁，浙江温州人，因“发现左耳后肿物2个月余”至我院就诊，术前凝血功能筛查示TT 22.4 s、Fg∶C 0.98 g/L、Fg∶Ag 1.01 g/L，其他凝血指标无明显异常。患者既往体健，肝肾功能正常，无自发性出血及血栓形成病史。其他家系成员（共2代3人）均无自发性出血及血栓形成病史。家系图见图2。

**图2:**
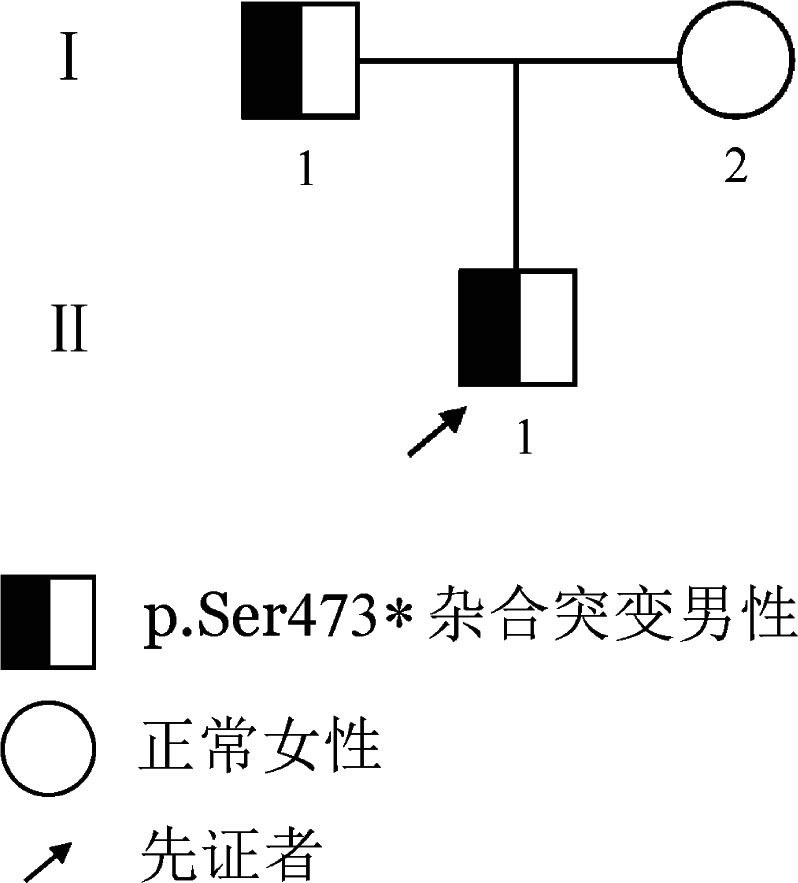
遗传性纤维蛋白原缺陷症（家系2）家系图

2. 健康对照组：以2022年11月在我院就诊的150名健康体检者作为健康对照组，用于排除基因多态性。其中男69名，女81名，中位年龄35（20～54）岁，均无肝肾功能异常，无出血及血栓病史，无口服抗凝药史。本研究通过本院伦理委员会审批（伦审2022-R193），所有受试者均签署知情同意书。

3. 标本采集与处理：采集所有研究对象外周静脉血2.7 ml，使用0.109 mol/L枸橼酸钠溶液1∶9抗凝，1 000×*g*离心10 min后取上层乏血小板血浆用于凝血指标检测及先证者纤维蛋白聚集试验，下层血细胞用于基因组DNA提取。

4. 凝血指标检测：应用法国Stago-STA-R-Max全自动血凝仪（法国Stago公司及配套试剂）采取凝固法测定凝血酶原时间（PT）、活化部分凝血活酶时间（APTT）、TT及Fg∶C，采取免疫比浊法测定D-二聚体（D-D）及纤维蛋白原降解产物（FDP）。应用LX20PRO全自动生化分析仪（美国Beckman Coulter公司）上采取免疫比浊法测定Fg∶Ag。应用全自动化学发光免疫分析仪Shine i2900（广州万孚生物技术有限公司）采取化学发光免疫分析法测定血浆凝血酶-抗凝血酶Ⅲ复合物（TAT）。所有操作步骤均遵循说明书要求进行。

5. 外周血基因组DNA提取及PCR扩增：采取血液样本基因组DNA提取试剂盒（购自北京天根生化科技有限公司）提取受试者全血基因组DNA。根据GenBank中FGA、FGB、FGG基因参考序列M64982、M64983、M10014，通过Primer primer5.0软件设计覆盖所有外显子及侧翼序列的引物，序列及扩增条件见文献[3]，引物由上海桑尼生物科技有限公司合成。PCR反应体系25 ml，包括Taq PCR Mastermix（美国Promega公司）12.5 µl，双蒸水7.5 µl，DNA模板3 µl，正向引物1 µl，反向引物1 µl。PCR反应步骤：95 °C预变性5 min，95 °C变性30 s，55 °C/56 °C退火30 s，72 °C延伸30 s，30个循环后，72 °C延伸10 min。扩增产物送上海桑尼生物科技有限公司进行测序。测序结果通过Chromas软件与美国NCBI数据库公布的人类纤维蛋白原基因参考序列进行比对，寻找突变位点。如发现基因突变位点进行反向测序加以证实，待确定突变位点后，再扩增其余家系成员相应外显子片段并进行测序分析。采取相同方法提取健康对照组的基因组DNA，并进行相应片段的PCR扩增及测序，用于排除基因多态性。

6. Western blot法检测血浆中FGB蛋白含量：先证者的血浆用磷酸缓冲液（PBS）以1∶20稀释，使用BCA法检测蛋白浓度，具体方法参照文献[4]。根据测定出的蛋白浓度，将正常人混合血浆及先证者的血浆计算配成相同浓度（2 µg/µl）。在非还原条件下，经10％十二烷基硫酸钠-聚丙烯酰胺凝胶电泳（SDS-PAGE）分离，转移至醋酸纤维素（PVDF）膜上，用5％脱脂牛奶封闭非特异性位点。一抗为小鼠抗人纤维蛋白原Bβ链IgG抗体（美国Proteintech Group公司），二抗为辣根过氧化物酶（HRP）标记的羊抗小鼠IgG抗体（上海碧云天生物技术有限公司）。与化学发光底物孵育后，用荧光图像分析仪（Luminescent Image Analyzer LAS-4000）检测。

7. 生物信息学特性分析：采用ClustalX-2.1-win软件将人类与美国NCBI（https://www.ncbi.nlm.nih.gov/homologene）公布的其余8种同源物种：黑猩猩（Pan troglodytes）、家犬（Canis lupus familiaris）、家牛（Bos taurus）、小家鼠（Mus musculus）、褐家鼠（Rattus norvegicus）、原鸡（Gallus gallus）、热带爪蟾（Xenopus tropicalis）、斑马鱼（Danio rerio）进行纤维蛋白原基因氨基酸序列比对，分析该突变氨基酸的保守性。利用Mutation Taster（https://www.mutationtaster.org/）、PolyPhen-2（http://genetics.bwh.harvard.edu/pph2/index.shtml）、Provean和SIFT（https://www.jcvi.org/research/provean）、LRT（http://varcards.biols.ac.cn/）在线进行突变位点致病性分析的预测。使用Swiss-pdb Viewer4.0.1软件对突变前后氨基酸空间结构及分子间作用力的改变进行分析（pdb ID: 3ghg）。

8. 纤维蛋白聚集试验：使用巴比妥缓冲液盐溶液（法国Stago公司）调节样本血浆Fg∶C为0.5 g/L，取稀释后血浆140 µl加入96孔板中，迅速加入人含钙凝血酶试剂（法国Stago公司）10 µl，使最终浓度为1NIH U/ml。使用Thermo ScientificVarioskan Flash全波长微孔板酶标仪，每间隔1 min测定一次血浆在350 nm处吸光度值，连续监测1 h。

## 结果

1. 先证者及其家系成员表型检测结果：家系1先证者（Ⅱ_3_）Fg∶C为1.28 g/L，Fg∶Ag为2.20 g/L，TAT升高至11.25 µg/L；其父亲（Ⅰ_1_）、姐姐（Ⅱ_2_）、弟弟（Ⅱ_5_）和女儿（Ⅲ_1_）Fg∶C均有不同程度降低，Fg∶Ag均正常，TAT有不同程度升高。家系2先证者（Ⅱ_1_）Fg∶C为0.98 g/L，Fg∶Ag为1.01 g/L，TAT正常；其父亲（Ⅰ_1_）Fg∶C为1.25 g/L，Fg∶Ag为1.10 g/L，TAT正常。两个家系其他成员的凝血指标无明显异常（表1）。

**表1:** 两个遗传性纤维蛋白原缺陷症家系表型检测结果

家系成员	PT（s）	APTT（s）	TT（s）	D-D（mg/L）	FDP（mg/L）	Fg∶C（g/L）	Fg∶Ag（g/L）	Fg∶C/Fg∶Ag	TAT（µg/L）
家系1									
先证者（Ⅱ_3_）	14.2	35.4	22.5	0.40	1.20	1.28	2.20	0.58	11.25
父亲（Ⅰ_1_）	14.3	36.1	23.4	0.45	1.70	1.15	2.11	0.55	10.72
哥哥（Ⅱ_1_）	13.2	36.3	17.8	0.33	2.35	2.53	2.72	0.93	3.81
姐姐（Ⅱ_2_）	12.9	35.5	25.5	0.50	1.30	1.29	2.30	0.56	11.79
丈夫（Ⅱ_4_）	13.5	35.2	18.3	0.41	2.22	3.53	3.62	0.97	2.58
弟弟（Ⅱ_5_）	13.6	39.2	24.6	0.37	1.80	1.22	2.33	0.52	10.67
妹妹（Ⅱ_6_）	12.8	32.8	16.3	0.37	2.28	2.61	2.78	0.94	2.10
女儿（Ⅲ_1_）	14.5	37.0	23.9	0.33	1.70	1.17	2.00	0.59	11.34
儿子（Ⅲ_2_）	12.2	36.7	15.5	0.35	2.88	3.21	3.42	0.94	1.98
家系2									
先证者（Ⅱ_1_）	13.1	31.4	22.4	0.15	1.00	0.98	1.01	0.97	2.73
父亲（Ⅰ_1_）	12.7	33.8	23.5	0.19	1.88	1.25	1.10	1.14	2.55
母亲（Ⅱ_2_）	12.5	35.4	17.3	0.20	2.22	2.93	3.24	0.90	1.83

参考值	11.5~14.6	29.0~43.0	14.0~20.0	<0.50	0~5.00	2.00~4.00	2.00~4.00	>0.70	<4.08

注 PT：凝血酶原时间；APTT：活化部分凝血活酶时间；TT：凝血酶时间；D-D：D-二聚体；FDP：纤维蛋白原降解产物；Fg∶C：纤维蛋白原活性；Fg∶Ag：纤维蛋白原抗原；TAT：血浆凝血酶-抗凝血酶Ⅲ复合物

2. 基因突变检测结果：家系1先证者检出FGB第2号外显子c.293C>A杂合错义突变（图3），导致98位丙氨酸突变为天冬氨酸（p.Bβ Ala98Asp），其父亲、姐姐、弟弟和女儿均检出该杂合错义突变，其哥哥、丈夫、妹妹和儿子为野生型。家系2先证者检出FGB第8号外显子c.1418C>G杂合无义突变（图3），导致473位丝氨酸突变为终止密码子（TGA），进而导致翻译提前终止，下游19个氨基酸缺失（p.Bβ Ser473*）；其父亲为杂合突变型，母亲为野生型。通过检索基因组聚合数据库（http://gnomad-sg.org/）、千人基因组数据库（http://www.1000genomes.org/）、多态性数据库（http://www.ncbi.nlm.nih.gov/snp/）及国内外有关文献，均未见p.BβAla98Asp及p.BβSer473*相关报道。以上两突变在健康对照组中未检出，排除了基因多态性可能。

**图3:**
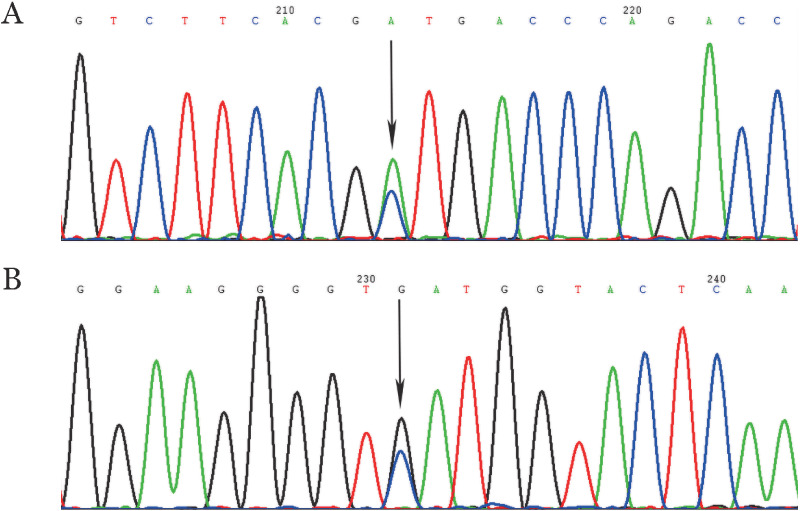
两个遗传性纤维蛋白原缺陷症家系测序结果（箭头所示为突变位点） A c.293C>A杂合错义突变正向测序结果； B c.1418C>G杂合无义突变正向测序结果

3. Western blot法检测血浆FGB蛋白含量：结果如图4所示，家系2先证者血浆FGB含量低于正常人混合血浆对照。

**图4:**
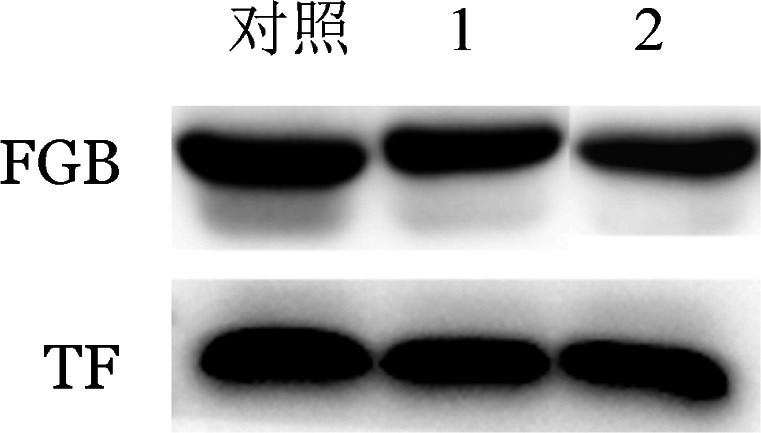
Western blot法检测两个遗传性纤维蛋白原缺陷症家系先证者血浆纤维蛋白原含量 对照：正常人混合血浆；TF：内参转铁蛋白；1和2分别为家系1先证者和家系2先证者

4. 突变氨基酸保守性分析：ClustalX-2.1-win软件保守性分析结果提示，p.BβAla98在9种同源物种中除家牛及原鸡外，呈高度保守性；p.BβSer473在9种同源物种间呈高度保守（图5）。

**图5:**
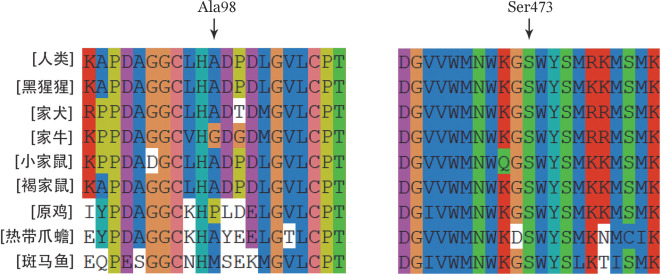
FGB基因同源物种多重序列比对结果

5. 生物信息学分析：五个在线生物信息学软件Mutation Taster、Provean、SIFT、Polyphen-2及LRT对c.293C>A突变的预测结果分别为“致病的”、“可容忍的”、“可容忍的”、“可能损害的”及“中立的”，得分分别为0.990、−1.740、0.056、0.859及0.001，提示该突变具有一定致病性。Mutation Taster及LRT对c.1418C>G的预测结果分别为“致病的”及“有害的”，得分为1.000和0.000，提示该突变存在一定的致病性，蛋白结构或功能改变的可能性较大。

6. 蛋白模型分析：使用Swiss-pdb Viewer4.0.1软件对突变前后蛋白模型进行分析，发现野生型Bβ链Ala98是非极性氨基酸，与带正电荷的极性氨基酸His97的苯环间形成一氢键，与带负电荷的极性氨基酸Asp99直接相连；当Ala98被Asp98取代后，与His97形成的氢键没有发生改变，而Asp98与Asp99之间形成一新的氢键。c.1418C>G突变蛋白模型预测结果显示该突变导致Ser473突变为终止密码子，最终产生一截短蛋白（Ser473*）（图6）。

**图6:**
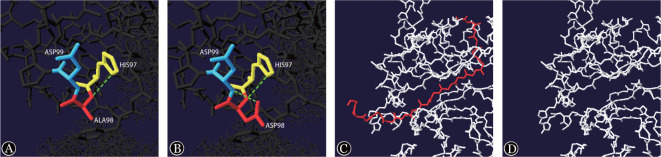
p. BβAla98Asp和p.BβSer473*突变前后蛋白模型分析图 A p.BβAla98Asp野生型； B p.BβAla98Asp突变型； C p.BβSer473*野生型； D p.BβSer473*突变型；绿色虚线代表氢键

7. 纤维蛋白聚集试验结果：家系1先证者纤维蛋白聚集曲线速率（聚集速度）及最大聚集率（峰值）与健康对照者相比明显降低；家系2先证者纤维蛋白聚集曲线与健康对照者相比无明显变化。

## 讨论

纤维蛋白原是一个由完全相同的两个亚基组成的共价二聚体，每个亚基包括Aα、Bβ、γ三条肽链，肽链间通过二硫键相互连接。位于4号染色体上的纤维蛋白原FGA、FGB、FGG基因分别编码Aα、Bβ、γ三条肽链，分别由610、461及411个氨基酸残基构成前体[5]。纤维蛋白原分子是一个对称性结构，包括由6条多肽链的N端组成中央的E区和两个由Bβ链及γ链的C端折回组成外围的D区，E区和D区之间由coiled-coil区相连[6]。纤维蛋白又称为凝血因子Ⅰ，在凝血、纤维蛋白溶解、伤口愈合、炎症、血管生成及肿瘤生长等方面发挥重要的作用[6]。纤维蛋白原Bβ链被认为是肝脏产生纤维蛋白原六聚体的限速因素[7]，因此FGB突变引起了广泛关注。

在本研究中，家系1先证者的Fg∶C明显降低，Fg∶Ag正常，为Ⅱ型缺陷；基因分析检出FGB第2号外显子错义突变p.BβAla98Asp，其家系中该杂合错义突变携带者均表现为Fg∶C不同程度降低而Fg∶Ag均正常。家系1先证者纤维蛋白聚集实验曲线图可见其纤维蛋白聚集功能下降。蛋白模型分析发现，p.BβAla98Asp错义突变后与周围氨基酸的氢键发生改变，导致纤维蛋白原组装及分泌发生变化。BβAla98位于高度保守的BβHis67-Pro70序列中，参与形成β转向。通过查阅相关文献发现同一位点已报道突变包括BβAla98Thr及BβAla98Ser。Koopman等[8]首次报道了fibrinogen Naples（p.BβAla98Thr），认为该突变影响凝血酶与纤维蛋白原结合，导致血液循环中游离活性凝血酶升高，进而引起血栓形成；其中的3名纯合突变携带者有卒中或血栓形成病史，而杂合突变携带者无临床症状。Yoshida等[9]报道了1例17岁日本男性上矢状窦血栓形成的纯合Ala98Thr病例。Ceznerová等[10]报道了1例轻度鼻出血的17岁女性，携带Ala98Ser杂合错义突变。Asp、Thr及Ser均为不带电荷的极性氨基酸，故而推测p.BβAla98Asp可能具有类似的致病机制，由于纤维蛋白原与凝血酶之间结合障碍，游离活性凝血酶升高，导致纯合突变携带者的血栓形成风险明显升高。该家系携带p.BβAla98Asp杂合突变的成员TAT水平均有不同程度升高，初步验证该突变位点可能导致纤维蛋白原与凝血酶结合障碍。

家系2先证者的Fg∶C与Fg∶Ag呈同步明显下降趋势，为I型缺陷。基因分析发现存在一个位于FGB第8号外显子上新的无义突变p.BβSer473*，其父亲为该杂合突变携带者，同样表现为Fg∶C与Fg∶Ag同步下降。血浆FGB蛋白Western Blot结果提示FGB含量较正常人降低。纤维蛋白聚集曲线图可见其聚集功能与健康对照者无明显差异。保守性分析结果提示该位点在同源物种间高度保守，在线生物信息学分析认为该突变为有害的突变。蛋白模型分析发现由于该无义突变导致终止密码子产生，翻译提前终止，进而导致其下游19个氨基酸缺失。BβSer473*位于Bβ链C端，是具有一个中心的五股β-片[11]。Casini等[12]认为Bβ链C端最后五个残基参与形成一个高度灵活的尾部，对疏水残基及亲水残基分布的细微变化非常敏感，因此作为保护屏障发挥防止水进入疏水核心及保持球体稳定性的作用，所以需正确折叠后才能向外分泌。Vu等[13]介绍了两种晚期无义突变Trp467*和TrpW470*分别从Bβ链C端最后去除了25个和22个残基，即表现出特异性损害纤维蛋白原分泌。为确定Bβ链哪些氨基酸是分泌纤维蛋白原所必需的，Vu等[13]构建了表达连续截断Bβ链的细胞模型，发现从Bβ链末端去除7个氨基酸（从Arg485）及以上，即可对纤维蛋白原六聚体的分泌产生影响。结合此前的文献报道可以推测，家系2先证者的纤维蛋白原Bβ链末端减少了19个残基，尾部的屏障作用减弱，导致稳定性下降，进而可能使纤维蛋白原含量降低。

综上所述，本文通过对两个CFD家系进行表型及基因型分析，发现了一个错义突变p.BβAla98Asp导致异常纤维蛋白原血症及一个无义突变p.BβSer473*导致低纤维蛋白原血症，但其具体分子致病机制有待进一步研究。
